# High-Accuracy Oral Squamous Cell Carcinoma Auxiliary Diagnosis System Based on EfficientNet

**DOI:** 10.3389/fonc.2022.894978

**Published:** 2022-07-07

**Authors:** Ziang Xu, Jiakuan Peng, Xin Zeng, Hao Xu, Qianming Chen

**Affiliations:** ^1^ State Key Laboratory of Oral Diseases, National Clinical Research Center for Oral Diseases, Chinese Academy of Medical Sciences Research Unit of Oral Carcinogenesis and Management, West China Hospital of Stomatology, Sichuan University, Chengdu, China; ^2^ Key Laboratory of Oral Biomedical Research of Zhejiang Province, Affiliated Stomatology Hospital, Zhejiang University School of Stomatology, Hangzhou, China

**Keywords:** oral squamous cell carcinoma, computational histopathology, deep learning, EfficientNet, auxiliary diagnosis

## Abstract

It is important to diagnose the grade of oral squamous cell carcinoma (OSCC), but the current evaluation of the biopsy slide still mainly depends on the manual operation of pathologists. The workload of manual evaluation is large, and the results are greatly affected by the subjectivity of the pathologists. In recent years, with the development and application of deep learning, automatic evaluation of biopsy slides is gradually being applied to medical diagnoses, and it has shown good results. Therefore, a new OSCC auxiliary diagnostic system was proposed to automatically and accurately evaluate the patients’ tissue slides. This is the first study that compared the effects of different resolutions on the results. The OSCC tissue slides from The Cancer Genome Atlas (TCGA, n=697) and our independent datasets (n=337) were used for model training and verification. In the test dataset of tiles, the accuracy was 93.1% at 20x resolution (n=306,134), which was higher than that at 10x (n=154,148, accuracy=90.9%) and at 40x (n=890,681, accuracy=89.3%). The accuracy of the new system based on EfficientNet, which was used to evaluate the tumor grade of the biopsy slide, reached 98.1% [95% confidence interval (CI): 97.1% to 99.1%], and the area under the receiver operating characteristic curve (AUROC) reached 0.998 (95%CI: 0.995 to 1.000) in the TCGA dataset. When verifying the model on the independent image dataset, the accuracy still reached 91.4% (95% CI: 88.4% to 94.4%, at 20x) and the AUROC reached 0.992 (95%CI: 0.982 to 1.000). It may benefit oral pathologists by reducing certain repetitive and time-consuming tasks, improving the efficiency of diagnosis, and facilitating the further development of computational histopathology.

## Introduction

Oral squamous cell carcinoma (OSCC) accounted for more than 377,713 new cancers and 177,757 deaths in 2020. The 5-year survival rate of patients in the earlier stage is about 55%–60%, while that of patients in advanced stages drops to 30%–40% (1, 2). The histological ‘grade’ of a malignant tumor is an index to describe its malignant degree. The current WHO classification of head and neck tumors is based on the simple grading system of the Broders standard (3), which is divided into three types: well-differentiated, moderately-differentiated, and poorly-differentiated. Later, more complex grading systems were suggested by Jakobsson et al. (4) and Anneroth et al. (5). The current way of diagnosing grades is still relying on the manual reading of slides by pathologists, which is a heavy workload, and the subjectivity of the pathologists greatly affects the diagnosis results, so it is valuable to develop an automatic auxiliary diagnosis system (6, 7).

Deep learning (DL) refers to the class of machine learning methods. It allows computers to learn complex concepts through relatively simple concepts (8). Since DL performs well in image interpretation and classification problems (9, 10), it has been widely used in medical image analysis tasks, especially in survival prediction and computational histopathology (11, 12), as well as classification of histological phenotypes (13).

Meanwhile, there have been several studies about the application of deep learning on the diagnosis of OSCC (10). For example, one study could judge whether the tissue is malignant (14, 15), but it could not determine the severity of the tumor tissue. Das et al. used only the images of the epithelial part to judge the grade of the tissue while the accuracy was not high enough (16). These studies have confirmed the application of deep learning in the field of OSCC, but there are still some imperfections, such as the lack of accuracy. Therefore, we carried out an automatic OSCC auxiliary diagnosis system, which was called EfficientNet-based Computational Histopathology of OSCC (ECHO). In this study, The Cancer Genome Atlas Program (TCGA) dataset was used to train and test the model (17). By comparing the performance of different convolutional neural networks (CNNs), the best performing one was be selected, and the performance was verified by using our independent dataset.

## Materials and Methods

**Figure 1:**
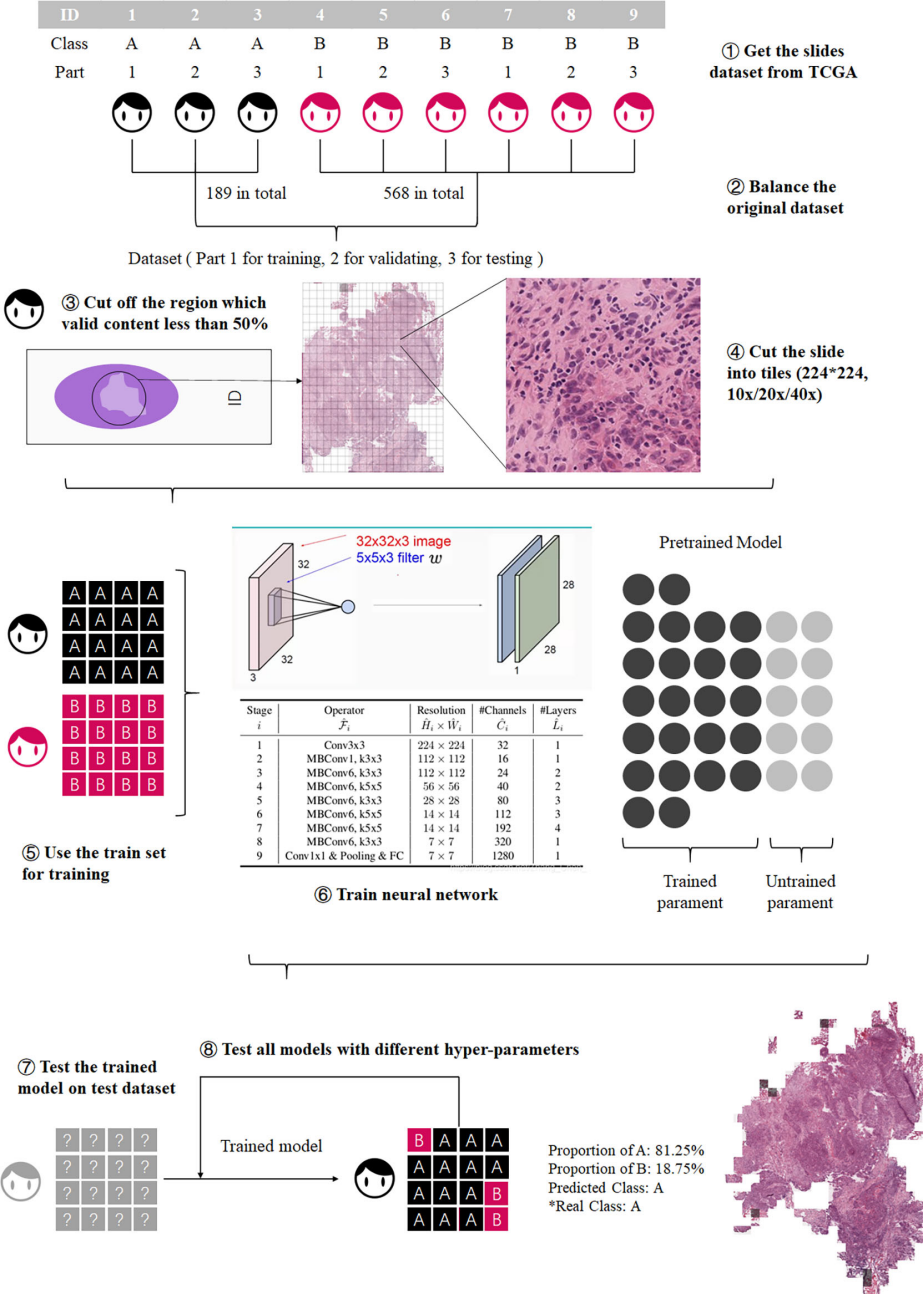
Process of model training. The training process was divided into three steps: preprocessing the datasets, model training, and model testing.

The workflow of this study is shown in **Figure 1**. First the slides were cut into tiles for training and testing (18), then the dataset was balanced by decomposing a multiclass imbalanced dataset into a binary problem (19), and the tiles with a blank area more than 50% were removed to ensure that each tile contains valid information. Then the preprocessed dataset was divided into training set, validation set, and test set.

Secondly, three CNNs, EfficientNet b0 (20), ShuffleNetV2 (21), and ResNeXt_18 (22), were trained at different resolutions, and the most accurate CNN with the best performing resolution was selected for the further analysis.

Finally, we tested the performance of ECHO on the external dataset, the OSCC tissue microarrays (TMA). There are differences in the image forms between TCGA and TMA, but both contain valid information, so we used TMA for external validation to prove that the model has high accuracy when dealing with various types of images.

The complete and detailed workflow is described below:

### Data Resource and Data Preprocessing

The image datasets include the TCGA OSCC image dataset and our independent TMA images dataset. We downloaded 757 whole-slide images of OSCC from the official website of TCGA in 2019 as the original dataset of TCGA. The TCGA dataset was used for model training and testing, and the TMA dataset was used for external verification. TCGA classifies OSCC into grade I (G1, well-differentiated), grade II (G2, moderately differentiated), grade III (G3, poorly differentiated), and grade IV (G4, undifferentiated or anaplastic) (23).

For the TCGA dataset, considering that the number of G4 slides was too small, and the imbalance of the dataset would seriously affect the training result, the G1 and G2 were combined as the well-differentiated group; G3 and G4 were combined as the poorly-differentiated group. There are 757 slides in the TCGA dataset, 568 slides in the G1-G2 group, and 189 slides in the G3-G4 group. These slides were cut into 224*224 pixel tiles (18, 20) at 10x, 20x, and 40x resolution, respectively, and the tiles with a blank area more than 50% were removed. The number of slides in the G3-G4 group and the G1-G2 group was quite different, which would adversely affect the results (19). We used the number of slides of the minimum class as the standard number, N0. Then calculated the ratio of N0 to the number of slides of each other class Nk. The ratio, Rk, was used to balance the tiles dataset. The tiles set of each class was multiplied by a coefficient Rk, as the final number of tiles for each class. The tiles of major classes were randomly removed until the numbers of tiles reached the final number. Then they were used as the preprocessed TCGA dataset.

The preprocessed data set was divided into three datasets: training set, validation set, and test set, which account for 60%, 20%, and 20% (24). These datasets were used for training and testing CNNs, and each CNN had two outputs: the possibility of G1-G2 and the possibility of G3-G4.

Additionally, we collected the TMAs dataset from the West China Hospital of Stomatology (Chengdu, China) and this study was approved by the ethics committee of the West China Hospital of Stomatology. The TMAs included 337 available slides of patients recruited from 2004 to 2014 who had received informed consent in this study. In the TMA dataset, according to the degree of tumor differentiation, the histological grades were divided into 1 (high differentiation), 2 (moderate differentiation), and 3 (low differentiation). Grades 1 and 2 were combined as the well-differentiated group (corresponding to G1-G2), and grade 3 was considered as the poorly-differentiated group (corresponding to G3-G4). The TMAs dataset was used for the external verification of the best performing CNN model chosen by above training steps. Moreover, they were used to verify the generalizability of the model.

### CNNs and Resolutions

For the consideration of training speed, training accuracy, and estimated time, we used three CNNs: EfficientNet b0, ShuffleNetV2, and ResNeXt_18. The performance of EfficientNet has shown great advantages since its inception, The accuracy and operation speed of EfficientNet is much faster than other networks (20), and it is often used as a comparison standard by the newly proposed CNNs (25, 26). ResNet is a classic neural network that is widely used in many fields and has good performance (22), so we chose ResNet as a benchmark to compare other CNNs. ShuffleNet is lightweight and computationally can be used on mobile devices (21). The reasons for choosing three models for this study was not only to select a better performing CNN, but also to try out the practicality of lightweight models.

These CNNs were trained on three different resolutions, and we selected the best CNN by comparing their accuracy and AUC on the tiles in test sets. In order to compare the effects of different resolutions on model training time and model accuracy, we decided to use 10x, 20x, and 40x resolution to train the three models separately. Finally, the model was used to evaluate the slides at the corresponding resolution. The resolution with better performance was selected.

### Model Training and Selection

In order to compare and select the best model more efficiently, all slides were cut into tiles which were used to train and test models. The label of a tile was determined by the slide which it came from. When the training was completed, the accuracy and AUC of the model on the tile dataset was used to evaluate the performance of the model, and in this way, the best model for the next study was selected.

Three networks were trained on the 10x resolution (154,148 tiles in all, 74,977 in G1-G2 group, 78,171 in G3-G4 group), 20x resolution (306,134 tiles in all, 149,826 in G1-G2 group, 156,308 in G3-G4 group), and 40x resolution (890,681 tiles in all, 469,751 in G1-G2 group, 420,930 in G3-G4 group). **Table 1** shows the information of the datasets used for training and testing.

**Table 1:** Datasets used for training and testing.

Dataset	G1-G2			G3-G4		
	10x	20x	40x	10x	20x	40x
Training	44,987	89,896	281,851	46,903	93,784	252,558
Validation	14,995	29,965	93,950	15,634	31,262	84,186
Test	14,995	29,965	93,950	15,634	31,262	84,186

We divided the tiles under each resolution into training, validation, and test sets in a ratio of 6:2:2.

During the training process, we observed the accuracy of each model at each epoch and drew an epoch-accuracy curve. When the epoch was low, the accuracy would also be low due to insufficient image feature extraction; when the epoch was high, the accuracy of the model would decrease due to over-fitting. When the epoch was around 60-70, the accuracy of the model would be high and stable (27). An epoch of about 60-70 would make the accuracy of the model high and stable, so the epoch was set to 80 and the accuracy of models was compared at each epoch.

Other hyperparameters are as follows: batch size: 80, learning rate: 0.0005, optimization algorithm: Adam, activation function: Swish.

### The Construction of ECHO

The best model and resolution selected in the above process was used to construct ECHO. Different from the above test process, the dataset here was composed of all slides. The accuracy on the slide dataset was used to evaluate the application value of ECHO.

The purpose of ECHO is to give the differentiation level of the input slide. The workflow mainly included two steps. First, ECHO cut the input slide into 224*224-pixel tiles and used the best model to give each tile a label of G1-G2 or G3-G4. In the second step, ECHO counted the tags of all tiles and used tags that account for more than 50% as the result of the slide. If the results given were consistent with the actual clinical labels, then ECHO’s prediction was considered accurate. The accuracy of ECHO’s predictions on all slides was used to evaluate the performance and application value of ECHO.

### Five-Classes Expansion of ECHO

Based on the best CNN selected by the above research and the most suitable resolution, we developed a five-class model of ECHO, which can assist the results of binary classification. The five classes are as follows: normal organization, G1, G2, G3, and G4. The data preprocessing and training process was the same as before. The effect of this model was evaluated by the confusion matrix and accuracy.

### Hardware and Software

Four NVIDIA Tesla K80 graphics cards were used, which contained a total of eight graphics processing units (GPUs). Each model was trained on a single GPU. The construction and training of the model was based on TensorFlow 2.1, and the programming language was Python 3.6.8.

### Statistical Analysis

We first got the accuracy and area under the receiver operating characteristic (ROC) curve (AUC) with 95% confidence interval (CI) in test datasets, then assessed the performance of networks. The 95% CI was calculated using the bootstrap method (28). The bootstrap method uses sampling with replacement, a sample size equal to the original sample size, and computes the required statistics. This process was repeated 100 times, and confidence intervals were estimated based on the statistics calculated for these 100 times. In our study, when calculating the accuracy, the original sample refers to whether the class of each whole-slide image (WSI) was judged correctly. When calculating AUC, the raw sample was the ratio of the actual label of each WSI to each class computed by the machine. The accuracy and AUC in test dataset were primary criteria for evaluation. All the statistical analysis was also performed with Python 3.6.8.

## Result

### Model Comparison

TCGA dataset was used to train and compare the performance of different CNNs and resolutions, then the best performing CNN and resolution were selected.

We first determined the epoch to be selected. In general, the accuracy of each model increases as the epoch grows. When the epoch reaches 50-60, the accuracy of the model has increased very little, and the difference was small. Therefore, the maximum value of the epoch was set to 80 and the accuracy of models was compared at each epoch. **Table 2** shows the epoch value of different models at three resolutions, and **Table 3** shows the accuracy and AUC at corresponding epoch values.

**Table 2:** The epoch value when the three models have the highest test accuracy in the three resolutions.

Model	Resolution		
	10x	20x	40x
EfficientNet	70	70	70
ShuffleNet	72	54	72
Resnet	76	78	64

We choose the epoch value with the highest accuracy as the parameter of the corresponding model. In the next test, we use the corresponding model to evaluate the effect.

**Table 3:** Accuracy and AUC of different CNNs and corresponding resolutions.

Model	Resolution		
	10x	20x	40x
EfficientNet	90.9 (0.97)	93.1 (0.98)	89.3 (0.96)
ShuffleNet	88.9 (0.96)	91.2 (0.96)	90.1 (0.97)
Resnet	76.1 (0.89)	90.8 (0.96)	89.8 (0.96)

Based on the best epoch value selected in **Table 2**, we tested the accuracy of different models and corresponding resolutions. The table shows the accuracy, with AUC values in parentheses. This result shows that EfficientNet at 20x resolution has the best performance.

**Figure 2:**
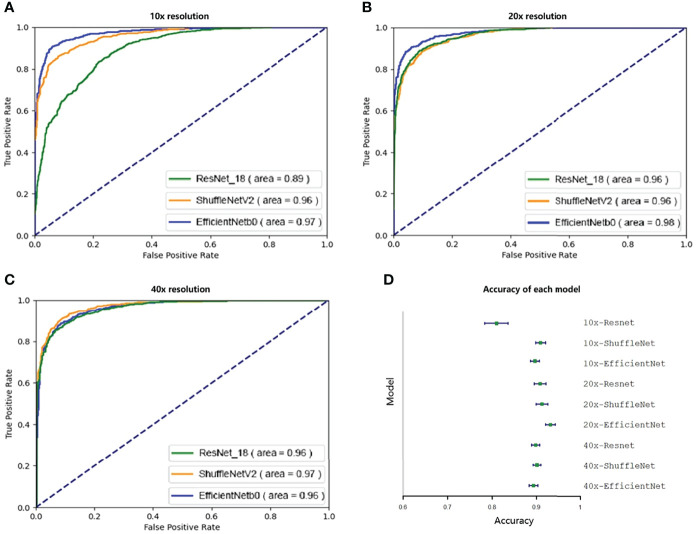
Ninety-five percent confidence interval when testing on a validation set of 10,000 tiles. The test set was resampled and tested one hundred times using the bootstrap method, and the ROC and 95% confidence interval were calculated. **A–C** show the ROC curves of three CNNs tested at 10x, 20x, and 40x resolution. Except for the 10x ResNet, the ROC of other models were all greater than 0.95. **D** shows the accuracy of each model. Except for 10x ResNet, the accuracy of each model was similar, while the accuracy of 20x EfficientNet is slightly higher.

Then we evaluated and selected the CNN and the resolution. **Figures 2A–C** shows the ROC of the three models at their best performance at three resolutions. Except for the 10x ResNet, the AUC of other models are all above 0.95, which maintained a high level. The highest among them was the EfficientNet at 20x. **Figure 2D** shows the accuracy of each CNN at different resolutions with 95% CI. The CNN with the highest accuracy was the EfficientNet at 20x, which accuracy reached 0.931 (95%CI: 0.920 to 0.942).

Because EfficientNet has better performance at 20x resolution both in accuracy and AUC, our next research will be based on this model, which is called ECHO.

**Figure 3:**
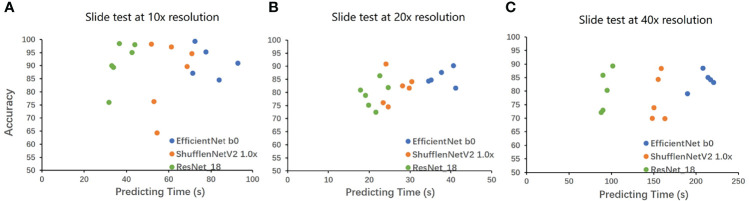
The result on the whole-slide-image. The WSIs was cut into tiles then classified, and the proportion of the correctly classified tiles was used as the accuracy to make Figure 3. The horizontal axis is the classification time (slide cutting time is omitted), and the vertical axis is the proportion of the correct classification. **A** is the result under 10x, **B** is the result under 20x, and **C** is the result under 40x. EfficientNet requires a longer time but has higher accuracy. ResNet has a very powerful speed and a good performance in accuracy. The speed of ShuffleNet is between the two, and the accuracy is not stable.

We also compared the calculation speed of different models. **Figure 3** shows the evaluation time and evaluation results of the three models for random whole-slide images. ResNet had the fastest computing speed. ShuffleNet took about 1.5 times that of ResNet, and EfficientNet took about 2 times that of ResNet. For larger slides, the time difference between the fastest and slowest models could be more than 60s, but the difference was acceptable in clinical practice.

### Test on the Whole-Slide Images

**Figure 4:**
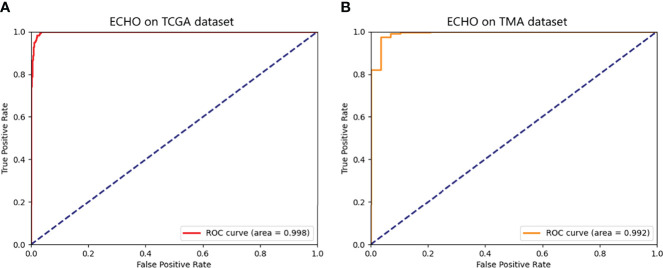
The ROC of the predicted results of ECHO. **A** shows the ROC tested and calculated by ECHO on the test set of TCGA. It can be seen that the area under the curve is as high as 0.998, with an accuracy of 98.1% (684/697). **B** shows the ROC tested and calculated by ECHO on TMA. The test accuracy rate is 0.914 (308/337), and the area under the curve is 0.992.

The ECHO was used to test on whole-slide image datasets of the TCGA dataset. If the proportion of G3-G4 tiles is more than 50%, the machine will judge the slide as G3-G4. If not, the slide will be classified as G1-G2. We tested a total of 697 slides, the sensitivity reached 98.3% (176/179) and the specificity reached 98.0% (508/518). The total accuracy reached 98.1% (95%CI: 97.1% to 99.1%), and the AUC reached 0.998 (95%CI: 0.995 to 1.000, **Figure 4A**). It proves that the ECHO has very high accuracy on the internal test set. The processing and classification time of a single WSI is about 30-60s (based on the size of the WSI, shown in **Figure 3B**)

### Verification on the External Dataset

The TMAs image dataset was used to verify the performance and external use of the ECHO. The TMAs dataset has a total of 337 slides. The slide dyeing method of TMA was different from that of TCGA, so the color characteristics of the image are different. Meanwhile, due to the different sources of patients, the histological structure of the tumor may also be slightly different. Both TCGA and TMA have the tissue which contains sufficient content for pathological diagnosis, and the processing methods are also consistent, so we used TMA to validate the mode to prove that the model has high accuracy when dealing with various types of images. Due to the differences between the TMAs dataset and the TCGA dataset, it was appropriate to use the TMAs dataset to verify the performance and external applicability of ECHO.

The accuracy reached 91.4% (95% CI: 88.4% to 94.4%), and the AUC was 0.992 (95%CI: 0.982 to 1.000). The ROC curve is shown in **Figure 4B**. This proves that the ECHO still has a good effect when faced with test subjects whose sources are quite different and have good applicability.

### Visualization of ECHO

**Figure 5:**
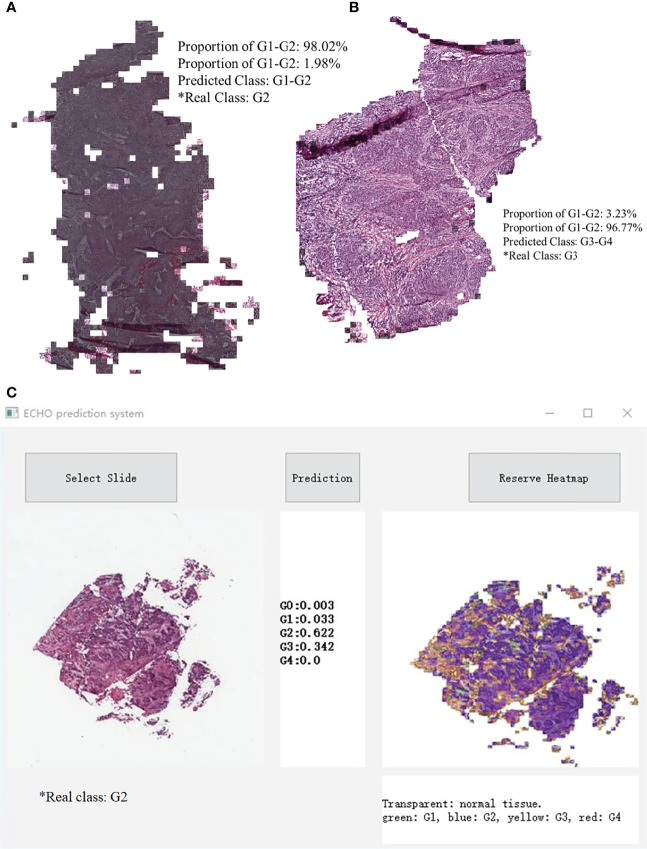
A slide image made in reverse according to the classification result. Most of the area in **A** is covered by black shadows, so this slide belongs to G1-G2. Picture **B** has almost no area covered by shadows, it belongs to G3-G4. **C** shows the classification results of the five classes ECHO program. The left area shows the input WSI preview, the middle area shows the probabilities of each category, and the right area shows the heatmap, each tile was added a color with transparency. Normal tissues, G1-G4 correspond to colorless, green, blue, yellow, and red, respectively.

When the prediction of each block was finished, the system made a restored slide picture according to the prediction result and the possibility of each block. If the predicted tile result was G1-G2, a black layer was added to the tile. We randomly selected two slides, which belong to G1-G2 and G3-G4. The result was stored through visualization, and the result is shown in **Figure 5**. **Figure 5C** shows the classification results of the five classes ECHO program. The right area shows the visualized heatmap, each tile was added color with transparency. Normal tissues, G1-G4 correspond to colorless, green, blue, yellow, and red, respectively. The detailed stored image can be seen in **Figure 1**, **2**.

### Evaluation of Five Classes ECHO

**Figure 6:**
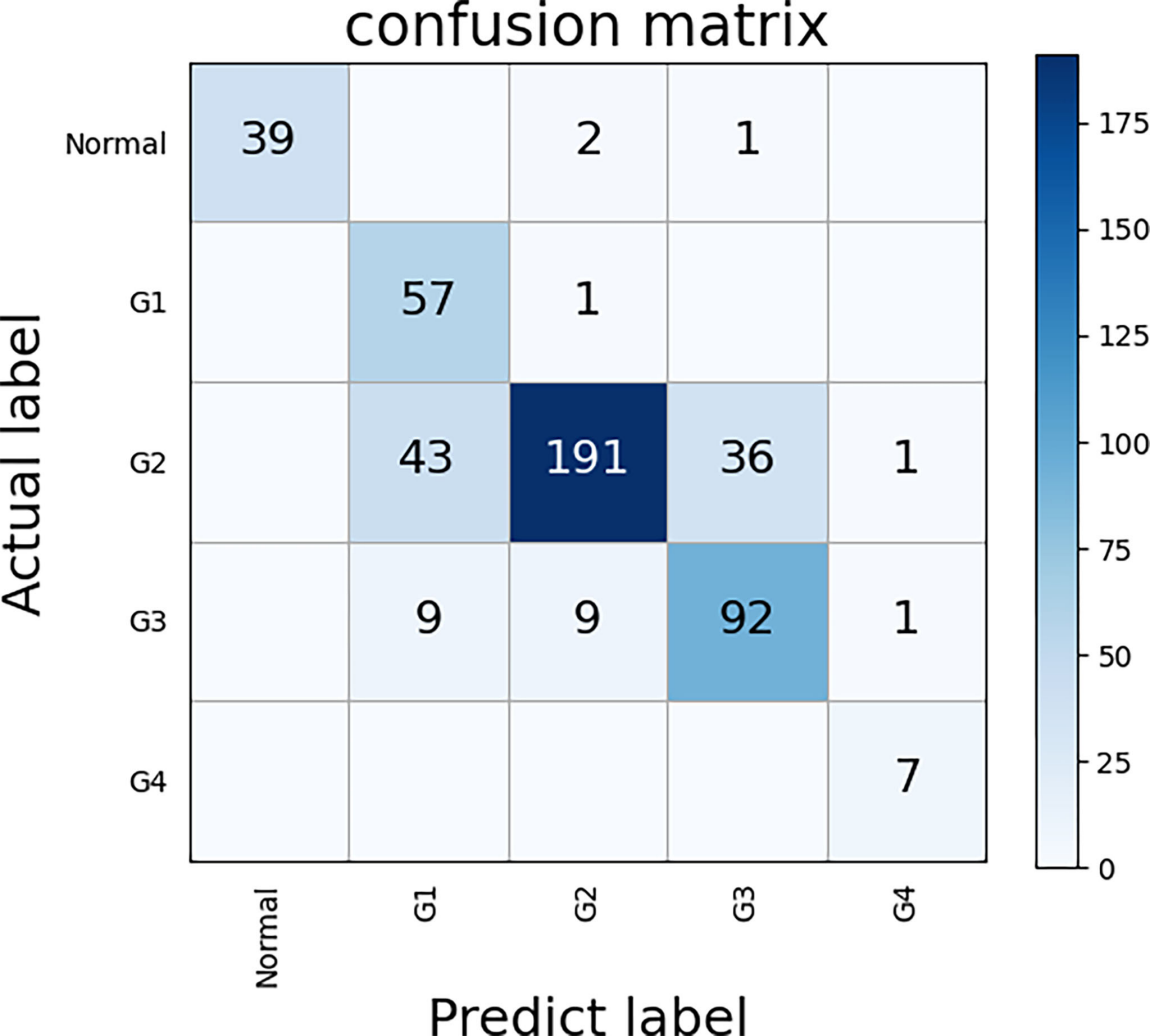
The confusion matrix of five classes ECHO. The correct prediction results are located on a diagonal line from the upper left corner to the lower right corner of the matrix. We used the bootstrap method to sample 100 times to calculate the confidence interval, and the final accuracy was 77.63% (95CI: 77.25-78.01).

We used the TCGA dataset to test the five classes ECHO. The five classes ECHO accepts a WSI as input and gives the probability of each class. In the test of 447 WSIs, 347 of them were correctly classified. We used the bootstrap method to sample 100 times to calculate the confidence interval, and the final accuracy was 77.63% (95CI: 77.25-78.01). **Figure 6** shows the confusion matrix according to the classification results. The results showed that the classification performance of different classes was different. The classification sensitivity of normal tissues, G1, and G4 were higher, reaching 92.85%, 98.27%, and 100%. The sensitivity of G3 and G2 was poor, 82.88% and 70.48%. In terms of specificity, the classification results of normal tissue and G2 were better, reaching 100% and 94.09%, G4, G3, and G1 are worse, being 77.78%, 71.31%, and 52.29%, respectively. The results show that the five classes ECHO can be used as a reference to complete some auxiliary tasks.

## Discussion

The visual inspection of tumor tissue under the light microscope by pathologists is the gold standard for OSCC grading This evaluation is mainly based on the pathologists’ clinical pathology knowledge and skills (29). The workload is heavy, and the results are affected by subjectivity. However, the application of DL in the histopathological diagnosis would help the pathologists (12, 30). Recently, there were several studies on OSCC automatic detection. For example, to judge whether OSCC is benign or malignant (14), CNNs were used to classify OSCC epithelial cells (16). However, these studies still have some imperfections, such as the methods used are relatively old, the data resources and evaluation indicators are single, and the accuracy is not high enough. In our study, these problems were basically solved. The ECHO achieved very high accuracy and verified the possibility of external application.

Firstly, we compared the performance of the three CNNs: EfficientNet, ShuffleNet, and ResNet. ShuffleNet is designed for mobile terminals (21), so the model has the smallest amount of parameters and the smallest size, which can be applied to lighter devices. As shown in **Figure 3**, its computing speed is at a medium level, and the highest accuracy reached 91.2% (95%CI: 89.9% to 92.5%, 20x resolution). The calculation speed of EfficientNet is the slowest in our study, but it was still faster than many CNNs (20). The accuracy of EfficientNet is the highest, which reached 93.1% (95%CI: 92.0% to 94.2%, 20x resolution). ResNet was a classic CNN that greatly alleviated the problem of overfitting (22). It has the fastest computing speed and has an accuracy of 90.8% (95CI: 89.5% to 92.1%, 20x resolution).

Secondly, we evaluated the impact of different resolutions on the experimental results. The higher resolution is helpful to improve the model’s recognition and extraction of image features, but it may affect the final result due to local overfitting (31). The reason may be that the cut tiles are too small and that the important features are at the edges, so that the key information cannot be extracted. Higher resolution will also greatly increase the workload of model training and the time of slide analysis. Lower resolution can effectively improve the training and recognition speed, but it may cause a potential decrease in accuracy. Because the resolution is too small, the details of the features are not clear, resulting in poor training results. Twenty times resolution also has faster application speed and accuracy, so this resolution was chosen to apply.

In this study, we took two measures to deal with imbalanced datasets. Since the number of slides in the G4 phase was too small, less than one-tenth of that in the G2 phase or G3 phase, we chose to combine G1 and G2 as well-differentiated, and G3 and G4 as poorly-differentiated. After the merger, the imbalance problem was alleviated. In the second step, we processed the dataset by undersampling the majority of class examples. To ensure that the information of each WSI can be utilized, we first cut all WSIs into tiles, and then undersampled the imbalanced tile set. The preprocessing measures we took may not be optimal, which leads to a loss of information (32). The undersampling process has produced good results, but in the next research, we will further explore better preprocessing measures (33).

There have been many studies on the machine learning application of OSCC. Mermod et al., 2020, used Random Forest (RF), linear Support Vector Machine (SVM), to judge the metastasis of squamous cell carcinoma of lymph nodes, with an accuracy of 90% (34). Romeo et al., 2020, used Naïve Bayes (NB), Bagging of NB, and K-Nearest Neighbors (KNN) to determine tumor grade with an accuracy of 92.9% (35). These researchers use more traditional machine learning methods, and there was still much room for improvement in accuracy. Ariji et al., 2019, who used deep learning methods, used CNN to evaluate lymph node metastasis, but the accuracy was only 78.1% (36). Jeyaraj & Samuel Nadar, 2019, used CNN to judge benign and malignant tumors with an accuracy of 91.4% (37). Our research is also based on CNN, which has two classification systems and five classification systems. The two-class classification system can accurately determine the tumor differentiation (high or low), and the accuracy has reached an astonishing 98.1%. The five-class classification system can judge the specific differentiation grade of the tumor and can also judge whether the tumor is malignant. The accuracy of judging whether it is benign or malignant has reached 92.86% (39/42). Therefore, our study is valuable and far surpasses other current studies in accuracy.

However, our research also needs improvement. Due to the limitation of the number of samples in the dataset, that is, the number of samples of G1, G2, G3, and G4 is too imbalanced, we had to group them to balance the amount of data. In future research, we will obtain more datasets to refine the model and train the system for five classes: normal, G1, G2, G3, and G4. In addition, in the division of G1-G2 and G3-G4, the machine determines whether a slide belongs to G1-G2 or G3-G4 according to the ratio of tiles. When the proportion of G3-G4 tiles is higher than 50%, the machine will classify this slide as ‘G3-G4’, so 50% is the threshold for machine judgment. It has been reported that when the threshold is 50%, the sensitivity is high and the specificity is low (38). When the threshold is changed, the effect of the model will be different, and this could be further discussed in the future.

## Conclusion

Oral squamous cell carcinoma is one of the most common head and neck tumors. It is important to determine the grade of tumor differentiation, which has a guiding role in tumor treatment and prognosis prediction. We developed two and five class systems based on CNN. The two classes system can judge whether the tumor is well differentiated or poorly differentiated. The test accuracy on the TCGA dataset reached 98.1% (n=697). The five classes system can judge whether the tissue belongs to normal tissue, G1, G2, G3, or G4. The accuracy reached 77.63%. We’ve also built visualization programs that can help doctors deal with some controversial slides. The system we developed can effectively reduce the workload of the pathologist and increase the efficiency and speed of the diagnostic process.

## Data Availability Statement

Publicly available datasets were analyzed in this study. This data can be found here: https://portal.gdc.cancer.gov.

## Ethics Statement

This study was approved by the ethics committee of the West China Hospital of Stomatology. The TMAs included 337 available slides of patients recruited from 2004 to 2014 who had received informed consent in this study.

## Author Contributions

HX, QC, ZX, and JP contributed to conception and design of the study. HX, ZX, XZ, and QC organized the database. ZX, JP, and HX performed the statistical analysis. ZX and JP wrote the first draft of the manuscript, JP, XZ, QC, and HX wrote sections of the manuscript. All authors contributed to manuscript revision, read, and approved the submitted version.

## Funding

This study was supported by grants from the National Natural Science Foundation of China (82001059).

## Conflict of Interest

The authors declare that the research was conducted in the absence of any commercial or financial relationships that could be construed as a potential conflict of interest.

## Publisher’s Note

All claims expressed in this article are solely those of the authors and do not necessarily represent those of their affiliated organizations, or those of the publisher, the editors and the reviewers. Any product that may be evaluated in this article, or claim that may be made by its manufacturer, is not guaranteed or endorsed by the publisher.

## Supplementary Material

Supplementary Figure 1 Another WSI visualization. The differentiation grade of this tissue is G2.

Supplementary Figure 2 A high-resolution image of the visualization results. G1 tiles are colored green, G2 tiles are colored blue, G3 tiles are colored yellow, and G4 tiles are colored red. Due to the influence of H&E staining, the actual color displayed by the G2 tile is purple.
