# Supplementary figures and images for: High-Accuracy Oral Squamous Cell Carcinoma Auxiliary Diagnosis System Based on EfficientNet
Source: Front Oncol. 2022 Jul 7;12:894978. doi: 10.3389/fonc.2022.894978 (PMC9302026; doi:10.3389/fonc.2022.894978)

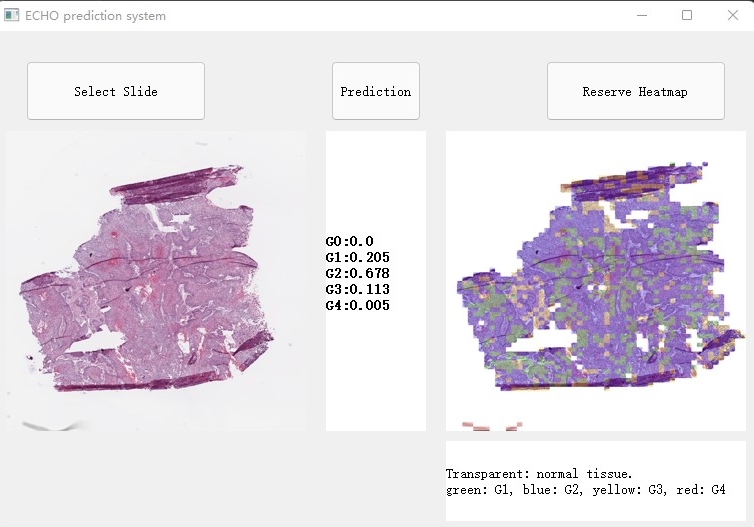

Supplement: Supplementary Figure 1 — Another WSI visualization. The differentiation grade of this tissue is G2. [file Image_1.jpeg]
